# Associations between smoke exposure and kidney stones: results from the NHANES (2007–2018) and Mendelian randomization analysis

**DOI:** 10.3389/fmed.2023.1218051

**Published:** 2023-08-10

**Authors:** Yong Huang, Hexi Wang, Chengwei Xu, Fulin Zhou, Huiyi Su, Yao Zhang

**Affiliations:** ^1^Department of Urology, The First Affiliated Hospital of Chongqing Medical University, Chongqing, China; ^2^Department of Respiratory Medicine, Children's Hospital of Chongqing Medical University, Chongqing, China

**Keywords:** smoking status, serum cotinine, kidney stone, NHANES, Mendelian randomization

## Abstract

**Purpose:**

It is currently controversial whether smoke exposure is associated with the risk of kidney stones. Herein, publicly available databases were combined to explore relationships with the risk of nephrolithiasis in terms of smoking status and serum cotinine concentrations.

**Materials and methods:**

First, we conducted an observational study using data from 2007 to 2018, based on the National Health and Nutrition Examination Survey (NHANES) database. Univariate analysis, multivariate logistic regression, trend testing, restricted cubic spline (RCS), and multiple imputation (MI) were the main analytical methods of our study. Then, A Mendelian randomization (MR) analysis was performed to explore the causal relationship between serum cotinine and nephrolithiasis. Genetic instruments for serum cotinine and pooled data for kidney stones were derived from publicly available large-scale genome-wide association studies (GWAS). Inverse-variance weighting (IVW) was the primary method for our MR analysis.

**Results:**

A total of 34,657 and 31,352 participants were included in the observational study based on smoking status and serum cotinine concentrations, respectively. Under full adjustment of covariates, current smokers had an increased risk of kidney stones compared to non-smokers [OR = 1.17 (1.04–1.31), *P* = 0.009, P for trend = 0.010]. Compared with serum cotinine of <0.05 ng/ml, serum cotinine levels of 0.05–2.99 ng/ml [OR = 1.15 (1.03–1.29), *P* = 0.013] and ≥3.00 ng/ml [OR = 1.22 (1.10–1.37), *P* < 0.001] were observed to have a higher risk of nephrolithiasis (*P* for trend < 0.001). In addition, a non-linear relationship between log2-transformed serum cotinine and the risk of nephrolithiasis was found (*P* for non-linearity = 0.028). Similar results were found when serum cotinine (log_2_ transformation) was used as a continuous variable [OR = 1.02 (1.01–1.03), *P* < 0.001] or complete data was used to analyze after MI. In the MR analysis, genetically predicted high serum cotinine was causally related to the high risk of nephrolithiasis [IVW: OR = 1.09 (1.00–1.19), *P* = 0.044].

**Conclusion:**

Current smoking and high serum cotinine concentrations may be associated with an increased risk of kidney stones. Further research is needed to validate this relationship and explore its underlying mechanisms.

## 1. Introduction

Kidney stones are a common urinary disorder characterized by the deposition of minerals that are free or attached to the renal papilla located in the renal pelvis or calyces (1). According to epidemiology surveys of kidney stones and published articles, the incidence and prevalence of kidney stones are increasing annually (2–4), which are influenced by many factors, such as sociodemographics, lifestyle habits, diseases, diet, and medications (5, 6). Early prevention of kidney stones can reduce the socioeconomic burden they cause (7, 8). Therefore, it makes sense to know the modifiable risk factors for kidney stones so that clinicians can better assist patients in preventing and treating kidney stones.

Previous studies have shown that smoke exposure is strongly associated with the risk of impaired health, including an increased risk of kidney stones (9, 10). One systematic review explored the relationship between lifestyle and nephrolithiasis, namely smoking, alcohol consumption, and exercise. The findings suggest a significant association between smoking and kidney stone formation, but further research is needed due to a lack of sufficient data (11). Cotinine, the most important primary metabolite of nicotine, is a biomarker of tobacco exposure, and its concentrations in the body are closely related to tobacco consumption (12, 13).

Nevertheless, there are few clinical studies discussing the association between smoke exposure and the risk of kidney stones. In this study, we explore the relationship between smoking status and kidney stone formation in terms of participants' smoking status and serum cotinine concentrations based on the National Health and Nutrition Examination Survey (NHANES). Mendelian randomization (MR) is a method of analysis through instrumental variables. It uses single nucleotide polymorphisms (SNPs) in genome-wide association studies (GWAS) as study objects to detect causality between exposure (e.g., serum cotinine) and outcome (e.g., kidney stone). Compared to observational studies, MR is not affected by confounding factors or reverse causation. Generally, our study combined the NHANES study and MR analysis to analyze the association between smoke exposure and the risk of nephrolithiasis, which would make the results more reliable.

## 2. Materials and methods

### 2.1. The study population in NHANES

**Figure 1:**
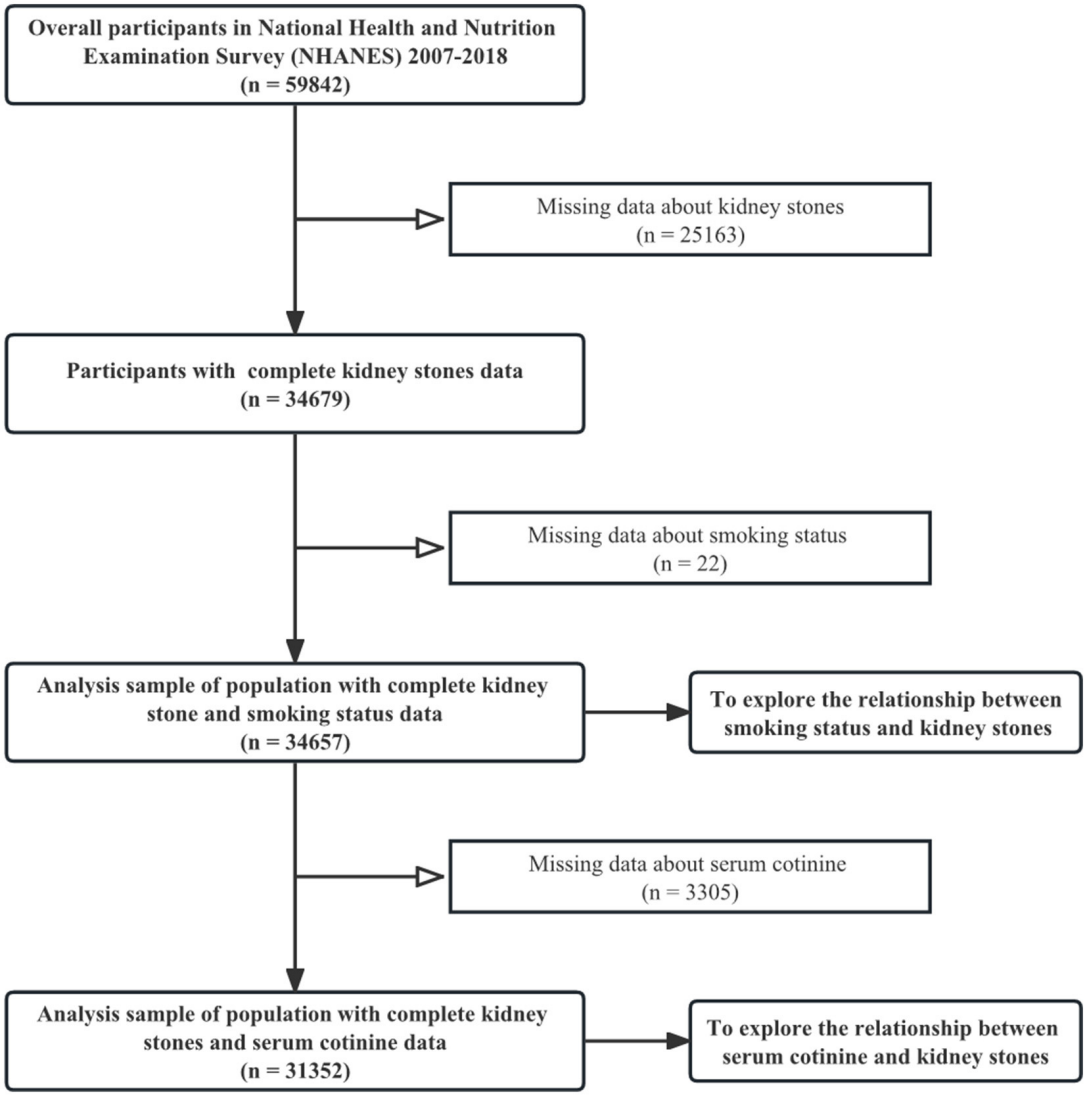
Flowchart of sample selection from NHANES (2007–2018).

The NHANES is a population-based cross-sectional survey designed to assess health and nutritional status within the US population (14). Since 1999, approximately 5,000 nationally representative individuals have been surveyed annually, and related data have been published biennially. Demographics, methodology, examination data, dietary data, questionnaire data, and laboratory data were included in the survey; specific information could be found on the website https://www.cdc.gov/nchs/nhanes. The National Center for Health Statistics (NCHS) Ethics Review Board approved the NHANES study protocol. In this study, data for six periods were included, from 2007 to 2018, in our collection. This period received attention because data on kidney stones became available in 2007 and the coronavirus disease 2019 (COVID-19) had not yet become epidemic in 2018. We explored the relationship between smoke exposure and kidney stones in terms of participants' smoking status and serum cotinine concentrations. The following were the exclusion criteria for this study: (1) missing/without data about kidney stones (*n* = 25,163); (2) missing/without data about smoking status (*n* = 22); and (3) missing/without data about serum cotinine concentrations in a population with a complete kidney stone and smoking status data (*n* = 3,305). Questionnaires on kidney stones were conducted only for those older than 20 years. Therefore, the population included in this study was all adults. In terms of smoking status, 34,657 participants out of 59,842 participants were screened in our study population. On the other hand, in terms of serum cotinine concentrations, a total of 31,352 participants were included. Figure 1 shows details of the inclusion and exclusion processes for this study.

### 2.2. Assessment of smoke exposure and kidney stones in NHANES

For the definition of smoking status, all participants needed to answer this question first, “In your lifetime, have you smoked more than 100 cigarettes?” Those who answered “No” were labeled as “Non-smokers,” while those who answered “Yes” were required to answer the following question further: “Do you smoke now?” The person who answered “Some days” or “Every day” was labeled as a “Current smoker,” and those who answered “Not at all” were labeled as “Former smokers.” In general, participants were divided into “non-smokers,” “former smokers,” and “current smokers.”

Cotinine, a major nicotine biomarker, was the first choice for assessing smoke exposure by measuring cotinine concentrations in blood, urine, and saliva (15). Compared with other diagnostic tools, cotinine was the best indicator of tobacco use because of its high sensitivity, good specificity, and long half-life (16). The liquid chromatography/atmospheric pressure ionization tandem mass spectrometric method was used to measure serum cotinine concentrations (17). Based on previous studies (18–20), we used 0.05 and 3.00 ng/ml as cutoff values to further convert serum cotinine concentrations into categorical variables. The class of serum cotinine was embodied in <0.05 ng/ml, 0.05–2.99 ng/ml, and ≥3.00 ng/ml. In addition, a log_2_ transformation was used for serum cotinine because of its skewed distribution, and then it was analyzed as a continuous variable.

Participants answered the question “Have you ever had kidney stones?” in the kidney condition questionnaire, and those who answered “Yes” were identified as having a clear history of kidney stones (4).

### 2.3. Covariate definition in NHANES

Through previously published studies, we screened factors associated with smoke exposure, cotinine, or kidney stones. These included age, sex, race, marital status, education, income-to-poverty ratio in the family (family PIR), body mass index (BMI), physical activity, serum uric acid, hypertension, coronary heart disease, diabetes, and gout (4, 21–25). Continuous variables included age, family PIR, BMI, and serum uric acid. Categorical variables included sex, race, marital status, education, physical activity, hypertension, coronary heart disease, diabetes, and gout. Vigorous or moderate work for at least 10 min continuously was labeled as an active physical activity. Hypertension was established when one of the following three conditions was present: systolic blood pressure ≥140 mmHg, diastolic blood pressure ≥90 mmHg or being on antihypertensive medications. Fasting blood glucose ≥126 mg/dl or glycohemoglobin ≥6.5% was labeled as diabetes. With or without coronary heart disease and gout were divided by answering the corresponding questionnaire “Yes” or “No.”

### 2.4. Genetically instrumental variables for serum cotinine in MR

**Figure 2:**
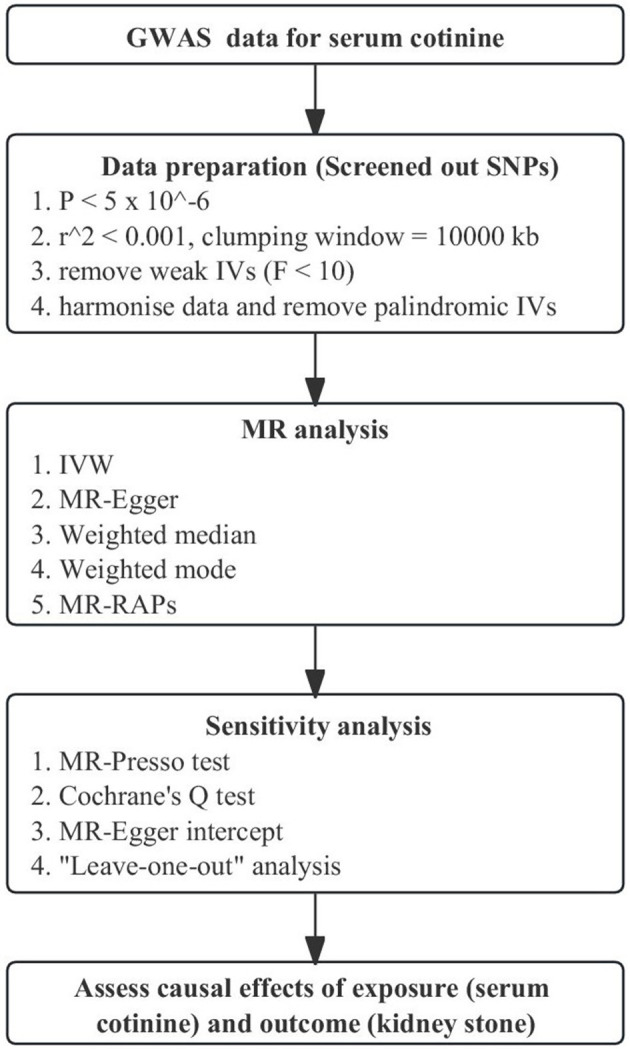
Flowchart of Mendelian Randomization analysis. MR, Mendelian randomization; GWAS, genome-wide association study; MR-PRESSO, MR-Pleiotropy RESidual Sum and Outlier; IVW, inverse-variance weighting; MR-RAPS, MR-robust adjusted profile score.

We used a genome-wide association study to obtain genetic instrumental variables for serum cotinine, which involved 5,185 current smokers (serum cotinine >10 ng/ml) of European ancestry (26). We screened the SNPs using the following steps, which were also the basic conditions of MR analysis: first, SNPs closely related to serum cotinine were screened out with a threshold *P* < 5 × 10^−6^. Then, under the parameters, *r*^2^ <0.001 and kb = 10,000, SNPs with linkage disequilibrium (LD) were removed. Finally, the *F* statistic was calculated for each SNP. Those *F* statistics >10 were considered strong instrumental variables (27) and strongly associated with serum cotinine. The flowchart of MR is shown in Figure 2. In addition, the three core assumptions about MR are shown in Supplementary Figure S1.

### 2.5. Genetic summary data on kidney stones in MR

GWAS summary data on kidney stones were available in the FinnGen consortium (https://finngen.gitbook.io/documentation/), which was a growing project among the Finns. We used the Release 6 (R6) version of data on kidney stones, which included 5,985 cases and 253,943 controls.

### 2.6. Statistical analyses

Complex sampling designs need to be considered to make our study population nationally representative. During the analysis, we used the sample weights from the NHANES database. By stratifying smoking status and serum cotinine concentrations, the weighted baseline characteristics of the overall population and participants under the corresponding stratification were described. Categorical variables and continuous variables were represented by percentages and the mean ± standard deviation (SD), weighted chi-square test, and weighted linear regression model were used to calculate the *P*-value, respectively. When analyzing serum cotinine concentrations as a continuous variable, a log_2_ transformation was implemented to meet a normal distribution. In addition, the variance inflation factor (VIF) was calculated to avoid the existence of multicollinearity, and a VIF of <10 could be considered to indicate that there was no multicollinearity between covariates (28). Univariate analysis was performed on participants based on smoking status and serum cotinine to judge the association between covariates and kidney stones. Then, three multivariate logistic regression models were constructed to explore the relationship between smoke exposure and kidney stones. Age, sex, race, and BMI were adjusted in Model 1. Model 2 adjusted marital status, education, family PIR, and physical activity based on Model 1. Model 3 adjusted serum uric acid, hypertension, coronary heart disease, diabetes, and gout based on Model 2. Based on Model 3 adjustments, a possible non-linear relationship was explored by constructing a restricted cubic spline (RCS) between serum cotinine (log_2_ transformation) and the risk of kidney stones. The number of knots in RCS was set to 5 because of the large sample size, and the serum cotinine concentration with OR = 1 was set as the reference value. In addition, to avoid bias in the results caused by too many missing values, the multiple imputation (MI) method, which was done *via* the “MICE” package in R software (29), was used to supplement the missing values and verify the robustness of the results. Five complete sets of data were obtained through MI, and finally, the effect values of the five datasets were integrated. Model 4 represented the effect values integrated after MI based on the fully adjusted model.

In this MR analysis, we used inverse-variance weighting (IVW) as our main research method. The presence or absence of heterogeneity determined whether a random-effects model or a fixed-effects model was used in the analysis. In addition, we used the MR-Egger, the weighted mode, and the weighted median for sensitivity analysis. Previous studies had described the details of these methods (30–32). The MR-Pleiotropy RESidual Sum and Outlier (MR-PRESSO) method was used to rule out outliers and test for horizontal pleiotropy. Furthermore, directional pleiotropy was determined by the MR-Egger intercept method. Heterogeneity was detected by the Cochrane Q test. MR-robust adjusted profile score (MR-RAPS) could correct pleiotropy with adjusted file scoring and make our results more reliable when many weak instrumental variables existed (31). Eventually, if the potential effects of SNPs were found in the “leave-one-out” analysis, then we needed to draw conclusions with caution (27). A scatter plot was created to visually observe the association between serum cotinine and the risk of nephrolithiasis.

All statistical analyses were performed using R Software (Version 4.2.1, http://www.R-project.org, The R Foundation) and Empowerstats Software (Version 2.0, http://www.empowerstats.com, X&Y Solutions, Inc., Boston, MA). In our study, a *p*-value of <0.05 was considered statistically significant.

## 3. Results

### 3.1. Baseline characteristics of the study participants in NHANES

In the aspect of smoking status, the weighted baseline characteristics of the study population are shown in Table 1. Non-smokers, former smokers, and current smokers consisted of 19,414 (56.02%), 8,219 (23.72%), and 7,024 (20.27%) participants, respectively. We found statistically significant differences in age, sex, race, marital status, education level, family PIR, BMI, physical activity, history of hypertension, history of coronary heart disease, history of diabetes, history of gout, and history of kidney stones in different smoking status groups (all *P*-values <0.001). The average age of the overall participants was 47.48 ± 17.02 (mean ± SD) years. The majority of the study population was female (51.91%) and non-Hispanic white (65.89%). Compared to the other groups, the current group of smokers was younger, had a lower family PIR, had lower serum uric acid, had a lower BMI, had active physical activity, and had no history of hypertension, coronary heart disease, diabetes, or gout. In particular, the population had a high prevalence of kidney stones (9.72%).

**Table 1:** Weighted baseline characteristics of participants with smoking status (*n* = 34,657).

**Characteristics**	**Smoking status**				***P*-value**
	**Overall**	**Non-smokers**	**Former smokers**	**Current smokers**	
Number of participants	34,657	19,414	8,219	7,024	-
**Kidney stone (%)**					<0.0001
No	90.19	91.13	87.97	90.28	
Yes	9.81	8.87	12.03	9.72	
**Sociodemographic variables**					
Age, years (mean ± SD)	47.48 ± 17.02	46.02 ± 17.10	54.37 ± 16.61	43.13 ± 14.66	<0.0001
Family PIR (mean ± SD)	2.98 ± 1.66	3.14 ± 1.65	3.17 ± 1.59	2.28 ± 1.58	<0.0001
**Sex (%)**					<0.0001
Male	48.09	41.80	57.56	54.23	
Female	51.91	58.20	42.44	45.77	
**Race (%)**					<0.0001
Mexican American	8.58	9.96	6.78	6.91	
Non-Hispanic Black	11.40	12.22	7.15	14.32	
Non-Hispanic White	65.89	61.41	75.56	66.66	
Other	14.13	16.41	10.51	12.11	
**Education (%)**					<0.0001
High school graduate or less	39.11	33.15	39.63	55.35	
Some college or AA	31.25	30.15	32.52	32.79	
College graduate or above	29.64	36.70	27.86	11.86	
**Marital status (%)**					<0.0001
Cohabitation	37.13	35.92	32.65	46.12	
Live alone	62.87	64.08	67.35	53.88	
**Laboratory data variables**					
Serum uric acid, mg/dl (mean ± SD)	5.42 ± 1.42	5.32 ± 1.40	5.68 ± 1.45	5.38 ± 1.39	<0.0001
**Physical examination and personal life**					
BMI, kg/m^2^ (mean ± SD)	29.08 ± 6.92	29.08 ± 6.99	29.81 ± 6.75	28.18 ± 6.80	<0.0001
**Physical activity (%)**					<0.0001
Active	44.91	41.59	46.26	52.65	
Inactive	55.09	58.41	53.74	47.35	
**Hypertension (%)**					<0.0001
No	65.73	68.78	54.75	70.84	
Yes	34.27	31.22	45.25	29.16	
**Diabetes (%)**					<0.0001
No	89.56	90.79	85.34	91.32	
Yes	10.44	9.21	14.66	8.68	
**Coronary heart disease (%)**					<0.0001
No	96.54	97.68	93.79	96.72	
Yes	3.46	2.32	6.21	3.28	
**Gout (%)**					<0.0001
No	95.93	96.84	92.96	97.03	
Yes	4.07	3.16	7.04	2.97	

As for grouping according to serum cotinine concentrations, the weighted baseline characteristics are shown in Table 2: 16,650 (53.11%), 6,426 (20.50%), and 8,276 (49.71%) participants in the group with serum cotinine of <0.05 ng/ml, 0.05–2.99 ng/ml, and ≥3.00 ng/ml, respectively. The average age of the overall participants was 47.57 ± 16.94 (mean ± SD) years. We did not observe a statistically significant difference in whether participants had a history of coronary heart disease (*P*-value = 0.176) or kidney stones (*P*-value = 0.067). The remaining characteristics in the serum cotinine ≥3.00 ng/ml group were similar to those of the participants in the current smoker group. Similarly, a high prevalence of kidney stones (10.19%) in participants with serum cotinine concentrations of ≥3.00 ng/ml was discovered.

**Table 2:** Weighted baseline characteristics of participants with serum cotinine concentrations (*n* = 31,352).

**Characteristics**	**Serum cotinine concentrations**				***P*-value**
	**Overall**	<**0.05 ng/ml**	**0.05–2.99 ng/ml**	≥**3.00 ng/ml**	
Number of participants	31,352	16,650	6,426	8,276	-
**Kidney stone (%)**					0.0672
No	90.08	90.41	89.45	89.81	
Yes	9.92	9.59	10.55	10.19	
**Sociodemographic variables**					
Age, years (mean ± SD)	47.57 ± 16.94	50.45 ± 16.97	45.19 ± 17.59	43.14 ± 15.10	<0.0001
Family PIR (mean ± SD)	2.99 ± 1.65	3.37 ± 1.58	2.68 ± 1.63	2.41 ± 1.61	<0.0001
**Sex (%)**					<0.0001
Male	48.21	43.12	48.70	58.68	
Female	51.79	56.88	51.30	41.32	
**Race (%)**					<0.0001
Mexican American	8.66	9.98	8.46	6.03	
Non-Hispanic black	10.76	6.93	15.93	15.24	
Non-Hispanic white	66.51	68.20	60.33	67.33	
Other	14.06	14.90	15.28	11.40	
**Education (%)**					<0.0001
High school graduate or less	38.82	29.74	44.96	53.75	
Some college or AA	31.38	29.38	34.68	33.28	
College graduate or above	29.80	40.88	20.36	12.97	
**Marital status (%)**					<0.0001
Cohabitation	36.55	30.01	43.90	45.21	
Live alone	63.45	69.99	56.10	54.79	
**Laboratory data variables**					
Serum uric acid, mg/dl (mean ± SD)	5.42 ± 1.42	5.32 ± 1.39	5.61 ± 1.47	5.48 ± 1.42	<0.0001
**Physical examination and personal life**					
BMI, kg/m^2^ (mean ± SD)	29.09 ± 6.89	29.01 ± 6.57	30.17 ± 7.65	28.48 ± 6.88	<0.0001
**Physical activity (%)**					<0.0001
Active	45.33	41.48	46.09	52.94	
Inactive	54.67	58.52	53.91	47.06	
**Hypertension (%)**					<0.0001
No	65.89	63.78	65.44	70.71	
Yes	34.11	36.22	34.56	29.29	
**Diabetes (%)**					0.0016
No	89.59	89.35	88.91	90.60	
Yes	10.41	10.65	11.09	9.40	
**Coronary heart disease (%)**					0.1763
No	96.52	96.35	96.81	96.68	
Yes	3.48	3.65	3.19	3.32	
**Gout (%)**					0.0392
No	95.94	95.92	95.47	96.33	
Yes	4.06	4.08	4.53	3.67	

### 3.2. Univariate analysis of kidney stones based on smoking status and serum cotinine concentrations in NHANES

The correlation between covariates and kidney stones based on smoking status is shown in Supplementary Table S1. Among participants with or without a history of kidney stones, we found that age, sex, race, marital status, BMI, education level, and serum uric acid were statistically significant (*P* < 0.001). On the other hand, hypertension, coronary heart disease, diabetes, and gout were closely related to the occurrence of kidney stones. The above features were also observed in participants based on serum cotinine concentrations, which are demonstrated in Supplementary Table S2.

### 3.3. Associations of smoking status and serum cotinine concentrations with the risk of kidney stones in NHANES

The association between smoking status and kidney stones is presented in Table 3. We found that the current smoker group was strongly associated with the occurrence of kidney stones compared to the non-smoker group regardless of the adjustment model [Model 1: OR = 1.19 (1.07–1.32), *P* < 0.001; Model 2: OR = 1.18 (1.05–1.31), *P* = 0.004; Model 3: OR = 1.17 (1.04–1.31), *P* = 0.009], while these features were not observed in former smokers (all *P* > 0.05). Similar results were obtained in former smokers (*P* = 0.249) and current smokers [OR = 1.16 (1.05–1.28), *P* = 0.005] in Model 4. Therefore, current smokers have a higher risk of developing kidney stones (*P* for trend in Model 1, Model 2, and Model 3: <0.001, 0.004, and 0.010, respectively).

**Table 3:** Association between smoking status, serum cotinine concentrations, and risk of kidney stones.

**Exposure**	**OR (95% CI)**, ***P***			
	**Model 1**	**Model 2**	**Model 3**	**Model 4**
**Smoking status, category**				
Non-smokers	Ref	Ref	Ref	Ref
Former smokers	1.08 (0.99, 1.18), 0.095	1.07 (0.97, 1.18), 0.182	1.06 (0.96, 1.17), 0.288	1.05 (0.96, 1.15), 0.249
Current smokers	1.19 (1.07, 1.32), <0.001	1.18 (1.05, 1.31), 0.004	1.17 (1.04, 1.31), 0.009	1.16 (1.05, 1.28), 0.005
*P* for trend	<0.001	0.004	0.010	-
**Serum cotinine concentrations**				
Log_2_-transformed serum cotinine (ng/ml)	1.02 (1.01, 1.03), <0.001	1.02 (1.01, 1.03), <0.001	1.02 (1.01, 1.03), <0.001	1.02 (1.01, 1.03), <0.001
**Cotinine level, category**				
<0.05 ng/ml	Ref	Ref	Ref	Ref
0.05–2.99 ng/ml	1.16 (1.04, 1.28), 0.005	1.16 (1.04, 1.30), 0.006	1.15 (1.03, 1.29), 0.013	1.15 (1.04, 1.28), 0.007
≥3.00 ng/ml	1.25 (1.14, 1.38), <0.001	1.25 (1.12, 1.39), <0.001	1.22 (1.10, 1.37), <0.001	1.23 (1.11, 1.36), <0.001
*P* for trend	<0.001	<0.001	<0.001	-

Model 1 adjusted age, sex, race, and BMI; Model 2 adjusted for Model 1 plus marital status, education, family PIR, and physical activity; Model 3 adjusted for Model 2 plus serum uric acid, hypertension, coronary heart disease, diabetes, and gout; Model 4 represented the effect values integrated after MI based on Model 3.

Table 3 shows the association between serum cotinine as a continuous or categorical variable and the occurrence of nephrolithiasis. Log2-transformed serum cotinine was linked with an increased risk of kidney stones [the effect values were the same for either model: OR = 1.02 (1.01–1.03), *P* < 0.001]. No matter which adjustment model they were under, those with serum cotinine of 0.05–2.99 ng/ml and ≥ 3.00 ng/ml had varying degrees of increased risk of developing kidney stones compared with the control group (serum cotinine <0.05 ng/ml), especially in participants with higher serum cotinine concentrations [≥3.00 ng/ml; Model 1: OR = 1.25 (1.14–1.38, *P* < 0.001; Model 2: OR = 1.25 (1.12–1.39), *P* < 0.001; Model 3: OR = 1.22 (1.10–1.37), *P* < 0.001; Model 4: OR = 1.23 (1.11–1.36), *P* < 0.001]. As the category of serum cotinine levels increased, the prevalence of kidney stones also increased (*P* for trend was all <0.001 in Model 1, Model 2, and Model 3). Effect values for each of the five complete sets of data after MI, based on smoking status and serum cotinine concentrations, are presented in Supplementary Tables S3, S4, respectively.

**Figure 3:**
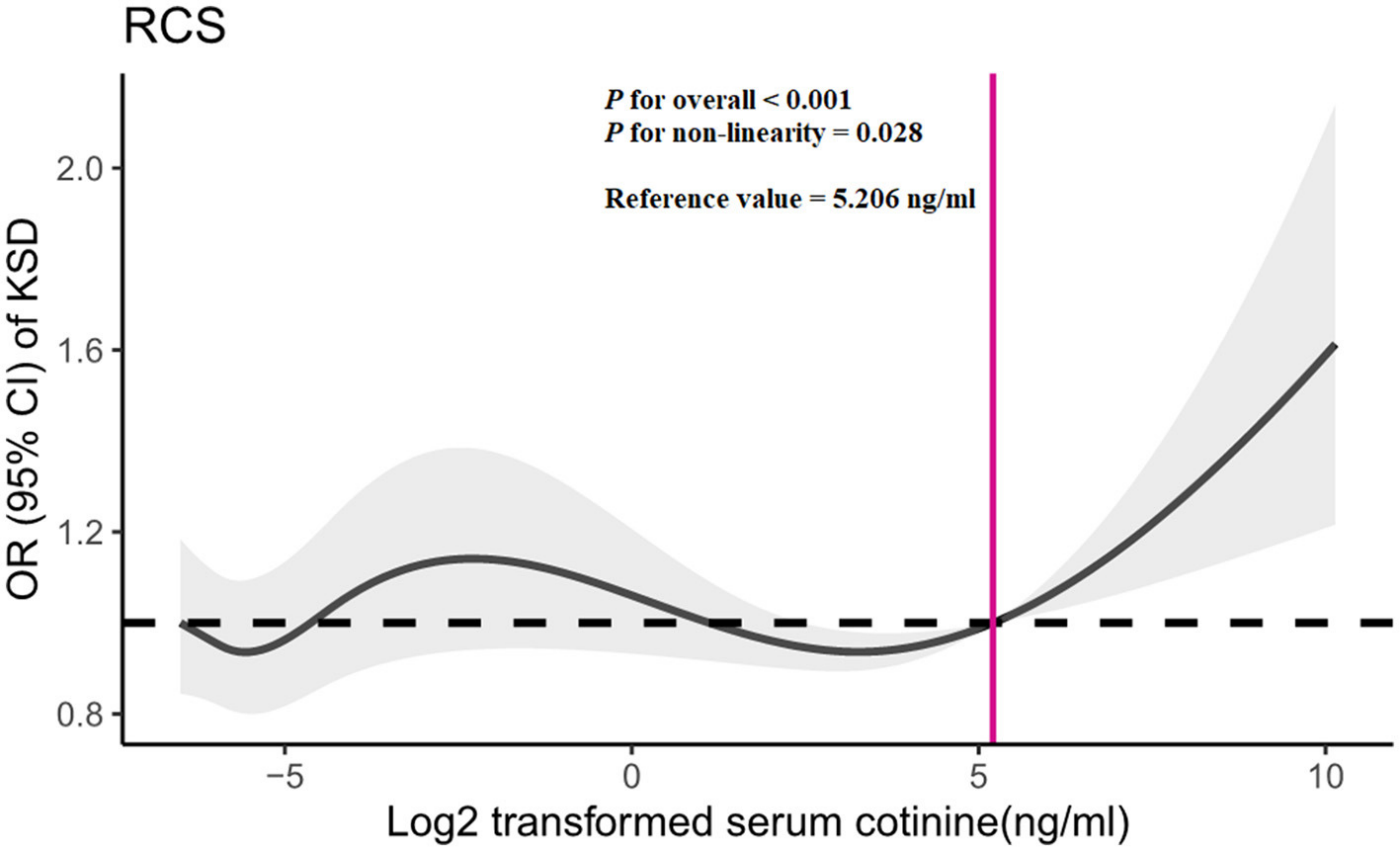
Restricted cubic spline (RCS) analysis of log2-transformed serum cotinine and odds ratio of kidney stone disease based on Model 3. OR, odds ratio; CI, confidence interval; KSD, kidney stone disease.

In RCS, we observed a non-linear relationship between log2-transformed serum cotinine and kidney stone risk (Figure 3; *P* for non-linearity = 0.028). The reference value is 5.206 ng/ml (serum cotinine = 36.91 ng/ml). When log_2_-transformed serum cotinine was below the reference value, the risk of kidney stones was little changed or even decreased, and when log_2_-transformed serum cotinine was above the reference value, the risk increased rapidly.

### 3.4. A causal association between serum cotinine and kidney stones in MR

**Figure 4:**
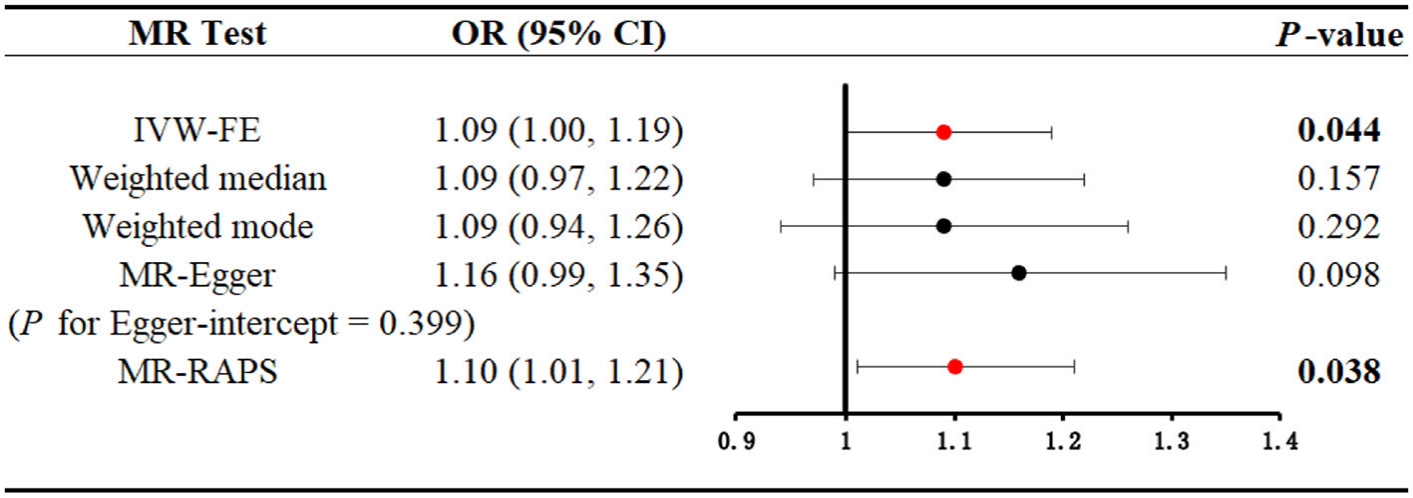
Mendelian randomization analysis to explore the causal association between serum cotinine and kidney stones. Bold and red dots indicate that there is statistical significance (*P* < 0.05). MR, Mendelian randomization; IVW-FE, inverse-variance weighting with fixed-effect model; MR-RAPS, MR-robust adjusted profile score.

**Figure 5:**
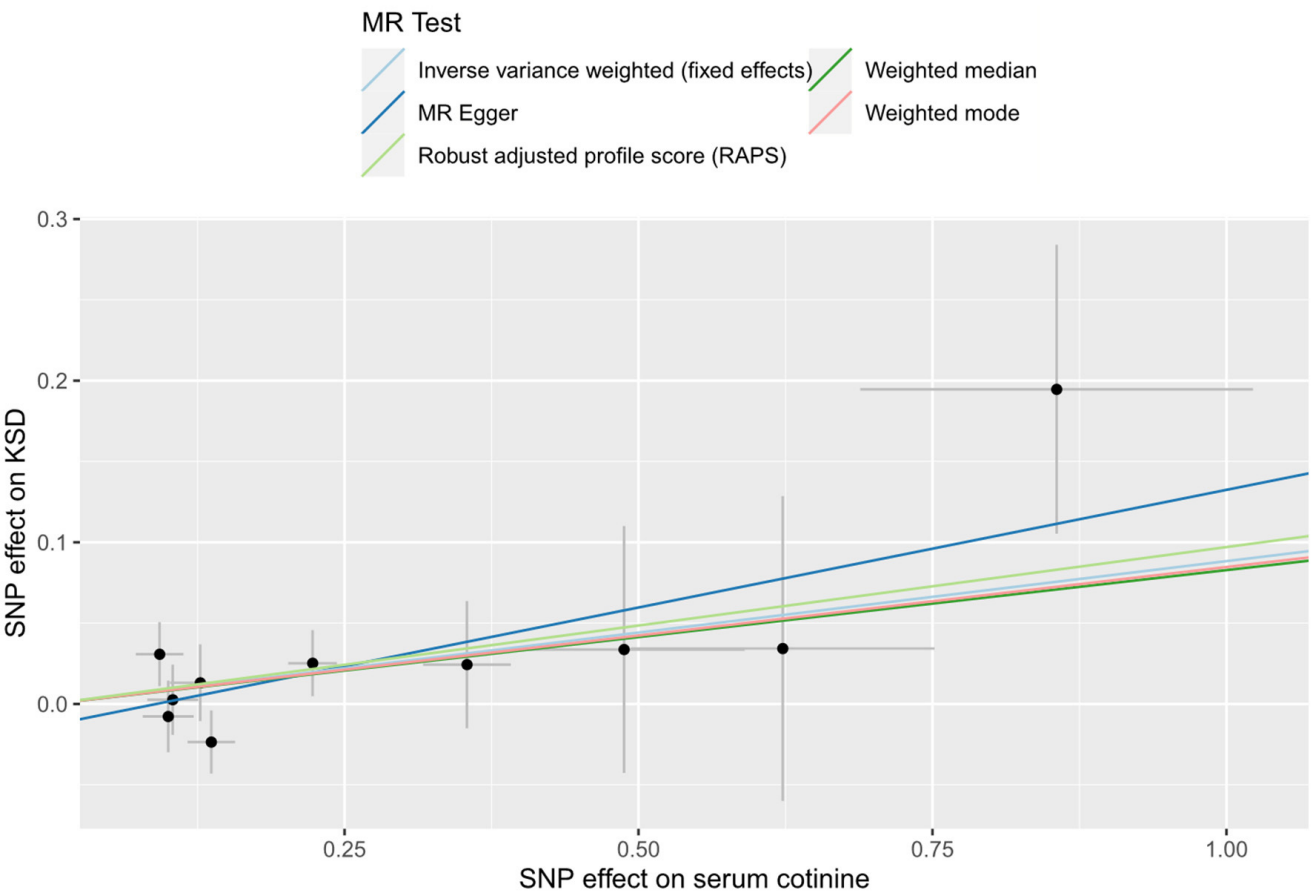
Scatter plot for THE causal effect of serum cotinine on kidney stones. MR, Mendelian randomization; SNP, single nucleotide polymorphism; KSD, kidney stone disease.

A total of 10 SNPs were eventually selected after a series of rigorous screenings, the details of which are shown in Supplementary Table S5. Genetically predicted serum cotinine was found to be associated with an increased risk of kidney stones by method IVW [Figure 4; OR = 1.09 (1.00–1.19), *P* = 0.044] and MR-RAPS [OR = 1.10 (1.01–1.21), *P* = 0.038], but the MR-Egger, the weighted mode, and the weighted median did not find a causal relationship between the two. Furthermore, heterogeneity, directional pleiotropy, and horizontal pleiotropy were not observed in this study (see Supplementary Table S6). The results of MR analysis were influenced by potential SNPs, as could be seen by the “leave-one-out” plot, so conclusions needed to be drawn with caution (Supplementary Figure S2). The scatter plot showed that as serum cotinine levels increased, so did the risk of nephrolithiasis, which is demonstrated in Figure 5.

## 4. Discussion

In this study, our results suggested smoke exposure as a risk factor for nephrolithiasis. The risk of kidney stones was increased in current smokers, which was found in our observational study. On the other hand, both the observational study and MR analysis supported a positive association between serum cotinine and the risk of nephrolithiasis. As far as we know, we were the first study to combine observational and MR studies to explore the relationship between smoke exposure and kidney stones.

A meta-analysis combining five observational studies published before 1 October 2021 showed a significant relationship between smoking and the risk of urolithiasis (33). In addition, later published research also found that both active and passive smoking may promote kidney stone formation (10, 11). However, a cross-sectional study of southeastern Iran found a significant association between smoking and the risk of nephrolithiasis, but this link disappeared after adjusting for other variables (34). In addition, a retrospective epidemiological study showed that there was no reliable evidence that smoking was associated with the occurrence and recurrence of kidney stones (35). As the association between smoking and nephrolithiasis is currently controversial, this article aims to evaluate the link between the two with the help of a publicly available database.

In this article, the NHANES and the publicly available GWAS database were utilized to combine observational studies and MR analysis, which aimed to reveal whether smoke exposure could lead to an increased risk of kidney stone formation. The observational study, which analyzed participants' smoking status and serum cotinine concentrations, found a significant association between current smokers and a high risk of kidney stones that did not exist in former smokers. As the results of the questionnaire were easily influenced, we added a more objective indicator, serum cotinine, which could reflect the extent of an individual's exposure to smoking (12). Similarly, serum cotinine was linked with an increased risk of nephrolithiasis both as a continuous variable (log_2_-transformed) and as a categorical variable. The above results were stable regardless of the adjustment model. In the MR analysis, our results supported the association of high serum cotinine concentrations with an increased risk of nephrolithiasis by the IVW and MR-RAPS methods. Five MR methods were used to assess causality. IVW was our primary method, and at least two MR methods supported a causal relationship between exposure (e.g., serum cotinine) and outcome (e.g., kidney stones) so that the results could be considered robust (36). Our observational study was conducted in the US population, and the GWAS data on serum cotinine and kidney stones for MR analysis were all from European populations. This may suggest to some extent that smoke exposure may increase the incidence of nephrolithiasis in different populations.

The exact mechanism by which smoke exposure causes kidney stones is currently unclear, but there are several possible explanations. First, tobacco smoke contains many harmful substances, such as cadmium and lead, and studies have shown that cadmium and lead concentrations in kidney stones are associated with smoking (37, 38). Cadmium and lead exposure may increase the risk of kidney stones (39, 40). Second, smoking may lead to an increase in vasopressin levels, which has a strong vasoconstrictive effect, causing a decrease in urine output (41). Low urine output was a common risk for all types of stones (21). Third, cigarette smoking was an independent risk factor for calcium urolithiasis, and it could reduce urinary calcium excretion or promote calcium deposition in the kidneys through a variety of mechanisms (35, 41). Furthermore, smoking could release reactive oxygen species (ROS), which could cause kidney damage and accelerate the development of chronic kidney disease (42, 43). Renal tubular damage or dysfunction could promote the nucleation, aggregation, and retention of crystals in the kidneys, eventually leading to the development of kidney stones (44). Further research is needed to explore and discover the underlying mechanisms of kidney stones caused by smoke exposure.

There are some merits to our study. First, we guaranteed a sufficiently large sample size based on the NHANES database. Second, the use of the MI method compensated for the influence of missing values, making the results more robust. Third, the RCS was constructed to explore the existence of a non-linear relationship. More importantly, we combined observational study and MR analysis. A causal inference could not be drawn from the NHANES study alone. MR analysis could compensate for the shortcomings of observational studies, which are susceptible to reverse causation and confounding factors. In addition, large-scale GWAS data were used for the MR analysis, so that it had sufficient statistical power to evaluate the relationship between smoke exposure and kidney stones.

However, there were also some limitations to our study. In the observational study, the information about smoking status and kidney stones was measured through questionnaires, and the results were easily influenced. Next, the data on kidney stones did not provide specific types of stones, so stratified analysis cannot be performed to identify how smoke exposure is related to specific types of stones. In addition, metabolic changes in women pre- and post-menopause may contribute to changes in the risk of kidney stones. Since pre- and post-menopausal data were only available for certain time periods (NHANES 2005–2010), further research was needed to explore the effects of smoking status and serum cotinine on the risk of kidney stones in pre- and post-menopausal women. In the MR analysis, sensitivity analysis did not yield consistent results. The possible influence of potentially confounding SNPs could not be ruled out. Finally, our study population was American and European, which made our results regionally limited.

## 5. Conclusion

The results of an observational study suggest that current smoking may increase the risk of kidney stones. Combining the observational studies and MR analysis, we found that high serum cotinine concentrations increased the risk of nephrolithiasis and were causally associated with kidney stones. This result needs to be confirmed by further research, and the underlying mechanisms still need to be explored.

## Data availability statement

The original contributions presented in the study are included in the article/Supplementary material, further inquiries can be directed to the corresponding author.

## Ethics statement

The Centers for Disease Control and Prevention (CDC) and the National Center for Health Statistics (NCHS) are responsible for conducting NHANES. All participants provided informed consent forms. The NHANES study protocol was approved by the NCHS Ethics Review Board. The GWAS data used in this study were derived from publicly published original studies that had received informed consent from participants and Ethics Committee approval at the time of publication.

## Author contributions

YH and YZ designed the research. YH conducted the formal analysis and wrote the original draft. HW and CX verified the data results. YH and HS made tables and figures. YH, HW, FZ, and HS edited and modified the article. YZ reviewed the final manuscript. All authors had read the final manuscript and endorsed its publication.

## Supplementary material

Supplementary Figure S1 Three core assumptions about Mendelian randomization.

Supplementary Figure S2 “Leave-one-out” analysis plot of the causal relationship between serum cotinine and kidney stones.

Supplementary Table S1 Univariate analysis of kidney stones in the study population based on smoking status.

Supplementary Table S2 Univariate analysis of kidney stones in the study population based on serum cotinine concentrations.

Supplementary Table S3 The respective and pooled effect values after multiple imputation (MI) are based on smoking status.

Supplementary Table S4 The respective and pooled effect values after multiple imputations are based on serum cotinine concentrations.

Supplementary Table S5 Detailed information about screened-out SNPs.

Supplementary Table S6 Sensitivity analysis of the Mendelian randomization study.

## References

[1] Khan SR Pearle MS Robertson WG Gambaro G Canales BK Doizi S. Kidney stones. Nat Rev Dis Prim. (2016) 2:16008. 10.1038/nrdp.2016.8 27188687 PMC5685519

[2] Scales CD Smith AC Hanley JM Saigal CS Urologic Diseases in America Project. Prevalence of kidney stones in the United States. Eur Urol. (2012) 62:160–5. 10.1016/j.eururo.2012.03.052 22498635 PMC3362665

[3] Tundo G Vollstedt A Meeks W Pais V. Beyond prevalence: annual cumulative incidence of kidney stones in the United States. J Urol. (2021) 205:1704–9. 10.1097/JU.0000000000001629 33502240

[4] Hill AJ Basourakos SP Lewicki P Wu X Arenas-Gallo C Chuang D. Incidence of kidney stones in the United States: the continuous national health and nutrition examination survey. J Urol. (2022) 207:851–6. 10.1097/JU.0000000000002331 34854755

[5] Stamatelou K Goldfarb DS. Epidemiology of kidney stones. Healthcare. (2023) 11:424. 10.3390/healthcare11030424 36766999 PMC9914194

[6] Chewcharat A Thongprayoon C Vaughan LE Mehta RA Schulte PJ O'Connor HM. Dietary risk factors for incident and recurrent symptomatic kidney stones. Mayo Clinic Proc. (2022) 97:1437–48. 10.1016/j.mayocp.2022.04.016 35933132

[7] Geraghty RM Cook P Walker V Somani BK. Evaluation of the economic burden of kidney stone disease in the UK: a retrospective cohort study with a mean follow-up of 19 years. BJU Int. (2020) 125:586–94. 10.1111/bju.14991 31916369

[8] Lotan Y Buendia Jiménez I Lenoir-Wijnkoop I Daudon M Molinier L Tack I. Primary prevention of nephrolithiasis is cost-effective for a national healthcare system. BJU Int. (2012) 110:E1060–1067. 10.1111/j.1464-410X.2012.11212.x 22686216

[9] Tamadon M R Nassaji M Ghorbani R. Cigarette smoking and nephrolitiasis in adult individuals. Nephrourol Mon. (2013) 5:702–5. 10.5812/numonthly.5251 23577335 PMC3614330

[10] Chen C-H Lee J-I Jhan J-H Lee Y-C Geng J-H Chen S-C. Secondhand smoke increases the risk of developing kidney stone disease. Sci Rep. (2021) 11:17694. 10.1038/s41598-021-97254-y 34489505 PMC8421344

[11] Jones P Karim Sulaiman S Gamage KN Tokas T Jamnadass E Somani BK. Do lifestyle factors including smoking, alcohol, and exercise impact your risk of developing kidney stone disease? Outc System Rev J Endourol. (2021) 35:1–7. 10.1089/end.2020.0378 32808537

[12] Hukkanen J Jacob P Benowitz N L. Metabolism and disposition kinetics of nicotine. Pharmacol Rev. (2005) 57:79–115. 10.1124/pr.57.1.3 15734728

[13] Chang CM Edwards SH Arab A Valle-Pinero AYD Yang L Hatsukami DK. Biomarkers of tobacco exposure: summary of an FDA-sponsored public workshop. Cancer Epidemiol Biomar Prev. (2017) 26:291–302. 10.1158/1055-9965.EPI-16-0675 28151705 PMC5336443

[14] Wang W Lu X Shi Y Wei X. Association between food insecurity and kidney stones in the United States: Analysis of the National Health and Nutrition Examination Survey 2007-2014. Front. Public Health. (2022) 10:1015425. 10.3389/fpubh.2022.1015425 36438222 PMC9682121

[15] Torres S Merino C Paton B Correig X Ramírez N. Biomarkers of exposure to secondhand and Thirdhand tobacco smoke: recent advances and future perspectives. Int J Environ Res Public Health. (2018) 15:2693. 10.3390/ijerph15122693 30501044 PMC6313747

[16] Raja M Garg A Yadav P Jha K Handa S. Diagnostic methods for detection of cotinine level in tobacco users: a review. J Clin Diagn Res. (2016) 10:ZE04–06. 10.7860/JCDR/2016/17360.7423 27135020 PMC4843405

[17] Bernert JT Turner WE Pirkle JL Sosnoff CS Akins JR Waldrep MK. Development and validation of sensitive method for determination of serum cotinine in smokers and nonsmokers by liquid chromatography/atmospheric pressure ionization tandem mass spectrometry. Clin Chem. (1997) 43:2281–91. 10.1093/clinchem/43.12.2281 9439445

[18] Benowitz NL Bernert JT Caraballo RS Holiday DB Wang J. Optimal serum cotinine levels for distinguishing cigarette smokers and nonsmokers within different racial/ethnic groups in the United States between 1999 and 2004. Am J Epidemiol. (2009) 169:236–48. 10.1093/aje/kwn301 19019851

[19] Benowitz NL Bernert JT Foulds J Hecht SS Jacob P Jarvis MJ. Biochemical verification of tobacco use and abstinence: 2019 update. Nicotine Tobacco Res. (2020) 22:1086–97. 10.1093/ntr/ntz132 31570931 PMC7882145

[20] Hou W Chen S Zhu C Gu Y Zhu L Zhou Z. Associations between smoke exposure and osteoporosis or osteopenia in a US NHANES population of elderly individuals. Front Endocrinol. (2023) 14:1074574. 10.3389/fendo.2023.1074574 36817605 PMC9935577

[21] Peerapen P Thongboonkerd V. Kidney stone prevention. Adv Nutr. (2023) 14:555–569. 10.1016/j.advnut.2023.03.002 36906146 PMC10201681

[22] Gao X Kong Y Li S Dong S Huang X Qi D. Intermediate effects of body mass index and c-reactive protein on the serum cotinine- leukocyte telomere length association. Front Aging Neurosci. (2021) 13:827465. 10.3389/fnagi.2021.827465 35115918 PMC8806079

[23] Feng X Wu W Zhao F Xu F Han D Guo X. Association between physical activity and kidney stones based on dose-response analyses using restricted cubic splines. Eur J Public Health. (2020) 30:1206–11. 10.1093/eurpub/ckaa162 32879977

[24] Weinberg AE Patel CJ Chertow GM Leppert JT. Diabetic severity and risk of kidney stone disease. Eur Urol. (2014) 65:242–7. 10.1016/j.eururo.2013.03.026 23523538 PMC3866968

[25] West B Luke A Durazo-Arvizu RA Cao G Shoham D Kramer H. Metabolic syndrome and self-reported history of kidney stones: the National Health and Nutrition Examination Survey (NHANES III) 1988-1994. Am J Kidney Dis. (2008) 51:741–7. 10.1053/j.ajkd.2007.12.030 18436084

[26] Buchwald J Chenoweth MJ Palviainen T Zhu G Benner C Gordon S. Genome-wide association meta-analysis of nicotine metabolism and cigarette consumption measures in smokers of European descent. Mol Psychiatry. (2021) 26:2212–23. 10.1038/s41380-020-0702-z 32157176 PMC7483250

[27] Wu F Huang Y Hu J Shao Z. Mendelian randomization study of inflammatory bowel disease and bone mineral density. BMC Med. (2020) 18:312. 10.1186/s12916-020-01778-5 33167994 PMC7654011

[28] Mela CF Kopalle PK. The impact of collinearity on regression analysis: the asymmetric effect of negative and positive correlations. Appl Econ. (2002) 34:667–77. 10.1080/00036840110058482

[29] Su YS Gelman A Hill J Yajima M. Multiple imputation with diagnostics (MI) in R: opening windows into the black box. J Stat Softw. (2011) 45:1–31. 10.18637/jss.v045.i02

[30] Bowden J Smith GD Haycock PC Burgess S. Consistent estimation in mendelian randomization with some invalid instruments using a weighted median estimator. Genet Epidemiol. (2016) 40:304–14. 10.1002/gepi.21965 27061298 PMC4849733

[31] Hartwig FP Davey Smith G Bowden J. Robust inference in summary data Mendelian randomization via the zero modal pleiotropy assumption. Int J Epidemiol. (2017) 46:1985–98. 10.1093/ije/dyx102 29040600 PMC5837715

[32] Bowden J Del Greco M F Minelli C Davey Smith G Sheehan NA Thompson JR. Assessing the suitability of summary data for two-sample Mendelian randomization analyses using MR-Egger regression: the role of the I2 statistic. Int J Epidemiol. (2016) 45:1961–74. 10.1093/ije/dyw220 27616674 PMC5446088

[33] Yue L Pai Q Wu X Zhang J. Smoking and risk of urolithiasis: meta-analysis of observational studies. Front. Public Health. (2022) 10:816756. 10.3389/fpubh.2022.816756 35321192 PMC8936389

[34] Khalili P Jamali Z Sadeghi T Esmaeili-Nadimi A Mohamadi M Moghadam-Ahmadi A. Risk factors of kidney stone disease: a cross-sectional study in the southeast of Iran. BMC Urol. (2021) 21:141. 10.1186/s12894-021-00905-5 34625088 PMC8499392

[35] Detsyk O Solomchak D. The impact of cigarette smoking, alcohol drinking and physical inactivity on the risk of urolithiasis occurrence and recurrence. Wiadomosci Lekarskie. (2017) 70:38–42. 28343191

[36] Ni J-J Xu Q Yan S-S Han B-X Zhang H Wei X-T. Gut microbiota and psychiatric disorders: a two-sample Mendelian randomization study. Front Microbiol. (2021) 12:737197. 10.3389/fmicb.2021.737197 35185808 PMC8856606

[37] Pappas RS. Toxic elements in tobacco and in cigarette smoke: inflammation and sensitization. Metallomics. (2011) 3:1181–98. 10.1039/c1mt00066g 21799956 PMC4542087

[38] Day PL Wermers M Pazdernik V Jannetto PJ Bornhorst JA. Detection of cadmium and lead in kidney stones. Associations with patient demographics, stone composition, and smoking. J Appl Lab Med. (2023) 8:330–40. 10.1093/jalm/jfac089 36575923

[39] Sun Y Zhou Q Zheng J. Nephrotoxic metals of cadmium, lead, mercury and arsenic and the odds of kidney stones in adults: An exposure-response analysis of NHANES 2007-2016. Environ Int. (2019) 132:105115. 10.1016/j.envint.2019.105115 31473411

[40] Huang J-L Mo Z-Y Li Z-Y Liang G-Y Liu H-L Aschner M. Association of lead and cadmium exposure with kidney stone incidence: A study on the non-occupational population in Nandan of China. J Trace Elements Med. Boil. (2021) 68:126852. 10.1016/j.jtemb.2021.126852 34508950

[41] Mooser V Burnier M Nussberger J Juillerat L Waeber B Brunner HR. Effects of smoking and physical exercise on platelet free cytosolic calcium in healthy normotensive volunteers. J Hypertens. (1989) 7:211–6. 10.1097/00004872-198903000-00007 2708817

[42] Jaimes EA Tian RX Raij L. Nicotine: the link between cigarette smoking and the progression of renal injury? Am J Physiol Heart Circ Physiol. (2007) 292:H76–82. 10.1152/ajpheart.00693.2006 16920799

[43] Mercado C Jaimes EA. Cigarette smoking as a risk factor for atherosclerosis and renal disease: novel pathogenic insights. Curr Hypertens Rep. (2007) 9:66–72. 10.1007/s11906-007-0012-8 17362674

[44] Khan SR. Renal tubular damage/dysfunction: key to the formation of kidney stones. Urol Res. (2006) 34:86–91. 10.1007/s00240-005-0016-2 16404622

