# Supplementary figures and images for: Associations between smoke exposure and kidney stones: results from the NHANES (2007–2018) and Mendelian randomization analysis
Source: Front Med (Lausanne). 2023 Aug 10;10:1218051. doi: 10.3389/fmed.2023.1218051 (PMC10450509; doi:10.3389/fmed.2023.1218051)

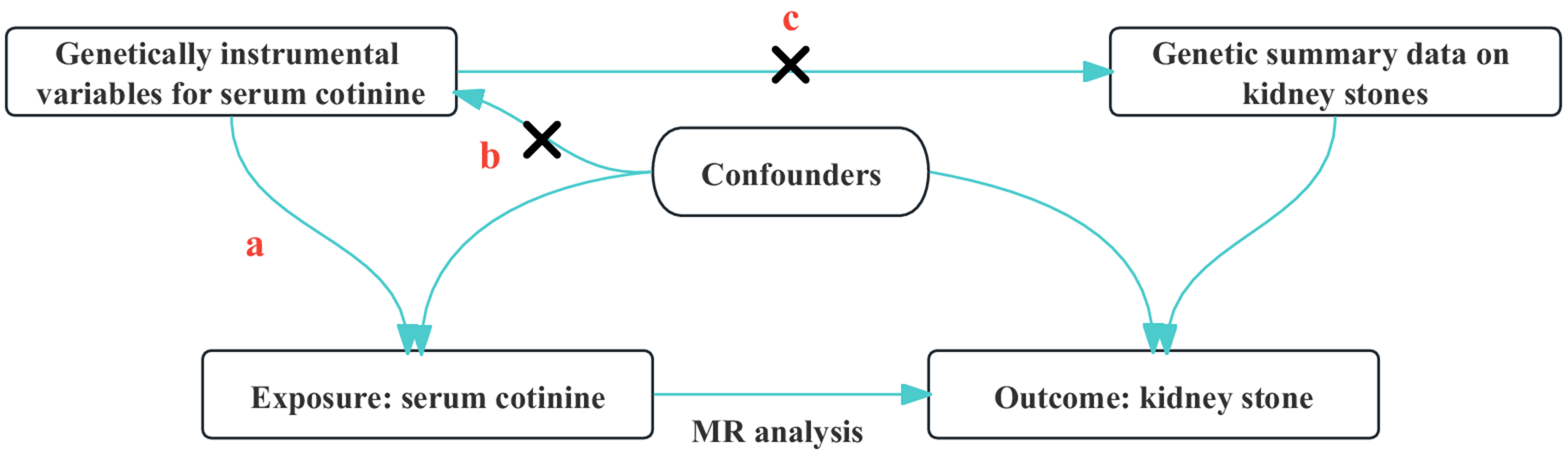

Supplement: Supplementary Figure S1 — Three core assumptions about Mendelian randomization. [file Image_1.PNG]
