# Supplementary material for: Associations between smoke exposure and kidney stones: results from the NHANES (2007–2018) and Mendelian randomization analysis
Source: Front Med (Lausanne). 2023 Aug 10;10:1218051. doi: 10.3389/fmed.2023.1218051 (PMC10450509; doi:10.3389/fmed.2023.1218051)
Supplement: Supplementary Table S1 — Univariate analysis of kidney stones in the study population based on smoking status. [file Table_1.DOCX]

**Supplementary** **Table S1.** Univariate analysis of kidney stones in the study population based on smoking status.

| Characteristics | Statistics | OR (95% CI), *P* |
| --- | --- | --- |
| Age, year (mean ± SD) | 47.48 ± 17.02 | 1.022 (1.020, 1.024), < 0.001 |
| Family PIR (mean ± SD) | 2.98 ± 1.66 | 0.99 (0.97, 1.02), 0.638 |
| BMI, kg/m^2^ (mean ± SD) | 29.08 ± 6.92 | 1.03 (1.02, 1.03), < 0.001 |
| Serum uric acid, mg/dl  (mean ± SD) | 5.42 ± 1.42 | 1.10 (1.07, 1.13), < 0.001 |
| Gender (%) | | |
| Male | 48.09 | 1 |
| Female | 51.91 | 0.75 (0.69, 0.80), < 0.001 |
| Race (%) | | |
| Mexican American | 8.58 | 1 |
| Non-Hispanic Black | 11.40 | 0.71 (0.62, 0.81), < 0.001 |
| Non-Hispanic White | 65.89 | 1.58 (1.42, 1.77), < 0.001 |
| Other | 14.13 | 1.02 (0.90, 1.16), 0.775 |
| Education (%) | | |
| High school graduate or less | 39.11 | 1 |
| Some college or AA | 31.25 | 1.03 (0.95, 1.12), 0.430 |
| Collage graduate or above | 29.64 | 0.85 (0.77, 0.93), < 0.001 |
| Marital status (%) | | |
| Cohabitation | 37.13 | 1 |
| Solitude | 62.87 | 1.20 (1.12, 1.30), < 0.001 |
| Physical activity (%) | | |
| Active | 44.91 | 1 |
| Inactive | 55.09 | 0.98 (0.91, 1.06), 0.611 |
| Hypertension (%) | | |
| No | 65.73 | 1 |
| Yes | 34.27 | 1.77 (1.64, 1.91), < 0.001 |
| Diabetes (%) | | |
| No | 89.56 | 1 |
| Yes | 10.44 | 1.85 (1.69, 2.03), < 0.001 |
| Coronary heart disease (%) | | |
| No | 96.54 | 1 |
| Yes | 3.46 | 2.30 (2.00, 2.64), < 0.001 |
| Gout (%) | | |
| No | 95.93 | 1 |
| Yes | 4.07 | 2.14 (1.87, 2.44), < 0.001 |
