# Supplementary material for: Associations between smoke exposure and kidney stones: results from the NHANES (2007–2018) and Mendelian randomization analysis
Source: Front Med (Lausanne). 2023 Aug 10;10:1218051. doi: 10.3389/fmed.2023.1218051 (PMC10450509; doi:10.3389/fmed.2023.1218051)
Supplement: Supplementary Table S2 — Univariate analysis of kidney stones in the study population based on serum cotinine concentrations. [file Table_2.DOCX]

**Supplementary Table S2.** Univariate analysis of kidney stones in the study population based on serum cotinine concentrations.

| Characteristics | Statistics | OR (95% CI), *P* |
| --- | --- | --- |
| Age, year (mean ± SD) | 47.57 ± 16.94 | 1.02 (1.02, 1.03), < 0.001 |
| Family PIR (mean ± SD) | 2.99 ± 1.65 | 1.00 (0.97, 1.02), 0.694 |
| BMI, kg/m^2^ (mean ± SD) | 29.09 ± 6.89 | 1.03 (1.02, 1.03), < 0.001 |
| Serum uric acid, mg/dl  (mean ± SD) | 5.42 ± 1.42 | 1.10 (1.07, 1.13), < 0.001 |
| Gender (%) | | |
| Male | 48.21 | 1 |
| Female | 51.79 | 0.72 (0.67, 0.78), < 0.001 |
| Race (%) | | |
| Mexican American | 8.66 | 1 |
| Non-Hispanic Black | 10.76 | 0.71 (0.61, 0.82), < 0.001 |
| Non-Hispanic White | 66.51 | 1.62 (1.44, 1.82), < 0.001 |
| Other | 14.06 | 1.01 (0.88, 1.15), 0.889 |
| Education (%) | | |
| High school graduate or less | 38.82 | 1 |
| Some college or AA | 31.38 | 1.07 (0.98, 1.16), 0.148 |
| Collage graduate or above | 29.80 | 0.86 (0.78, 0.95), 0.002 |
| Marital status (%) | | |
| Cohabitation | 36.55 | 1 |
| Solitude | 63.45 | 1.20 (1.11, 1.29), < 0.001 |
| Physical activity (%) | | |
| Active | 45.33 | 1 |
| Inactive | 54.67 | 0.99 (0.91, 1.07), 0.731 |
| Hypertension (%) | | |
| No | 65.89 | 1 |
| Yes | 34.11 | 1.77 (1.63, 1.91), < 0.001 |
| Diabetes (%) | | |
| No | 89.59 | 1 |
| Yes | 10.41 | 1.86 (1.70, 2.04), < 0.001 |
| Coronary heart disease (%) | | |
| No | 96.52 | 1 |
| Yes | 3.48 | 2.32 (2.01, 2.69), < 0.001 |
| Gout (%) | | |
| No | 95.94 | 1 |
| Yes | 4.06 | 2.18 (1.90, 2.50), < 0.001 |
