# Supplementary material for: Associations between smoke exposure and kidney stones: results from the NHANES (2007–2018) and Mendelian randomization analysis
Source: Front Med (Lausanne). 2023 Aug 10;10:1218051. doi: 10.3389/fmed.2023.1218051 (PMC10450509; doi:10.3389/fmed.2023.1218051)
Supplement: Supplementary Table S3 — The respective and pooled effect values after multiple imputation (MI) are based on smoking status. [file Table_3.DOCX]

**Supplementary** **Table S3.** The respective and pooled effect values after multiple imputation based on smoking status.

| **Smoking status** | **Model 1**  **OR (95% CI), *P*** | **Model 2**  **OR (95% CI), *P*** | **Model 3**  **OR (95% CI), *P*** |
| --- | --- | --- | --- |
| **Dataset 1** | | | |
| Non smoker | 1 | 1 | 1 |
| Former smoker | 1.082 (0.990, 1.183), 0.08159 | 1.068 (0.977, 1.168), 0.14769 | 1.054 (0.963, 1.153), 0.25171 |
| Current smoker | 1.192 (1.080, 1.316), 0.00051 | 1.161 (1.048, 1.286), 0.00420 | 1.155 (1.042, 1.279), 0.00585 |
| *P* for trend | < 0.001 | 0.003 | 0.006 |
| **Dataset 2** | | | |
| Non smoker | 1 | 1 | 1 |
| Former smoker | 1.082 (0.990, 1.183), 0.08366 | 1.068 (0.976, 1.168), 0.15062 | 1.054 (0.963, 1.153), 0.25607 |
| Current smoker | 1.192 (1.080, 1.317), 0.00050 | 1.163 (1.050, 1.288), 0.00370 | 1.159 (1.046, 1.284), 0.00469 |
| *P* for trend | < 0.001 | 0.003 | 0.005 |
| **Dataset 3** | | | |
| Non smoker | 1 | 1 | 1 |
| Former smoker | 1.084 (0.992, 1.185), 0.07591 | 1.070 (0.979, 1.170), 0.13670 | 1.056 (0.966, 1.156), 0.23094 |
| Current smoker | 1.194 (1.081, 1.318), 0.00046 | 1.170 (1.057, 1.296), 0.00255 | 1.167 (1.053, 1.292), 0.00317 |
| *P* for trend | < 0.001 | 0.002 | 0.004 |
| **Dataset 4** | | | |
| Non smoker | 1 | 1 | 1 |
| Former smoker | 1.082 (0.990, 1.183), 0.08349 | 1.068 (0.976, 1.168), 0.15096 | 1.054 (0.963, 1.153), 0.25276 |
| Current smoker | 1.193 (1.081, 1.318), 0.00047 | 1.164 (1.051, 1.290), 0.00348 | 1.158 (1.046, 1.283), 0.00486 |
| *P* for trend | < 0.001 | 0.003 | 0.005 |
| **Dataset 5** | | | |
| Non smoker | 1 | 1 | 1 |
| Former smoker | 1.083 (0.991, 1.184), 0.07974 | 1.068 (0.977, 1.168), 0.14941 | 1.054 (0.964, 1.154), 0.24764 |
| Current smoker | 1.189 (1.077, 1.313), 0.00062 | 1.157 (1.045, 1.282), 0.00509 | 1.151 (1.039, 1.275), 0.00702 |
| *P* for trend | < 0.001 | 0.004 | 0.007 |
| **Integration of effect values for five datasets** | | | |
| Non smoker | 1 | 1 | 1 |
| Former smoker | 1.083 (0.990, 1.183), 0.080554 | 1.068 (0.977, 1.168), 0.147217 | 1.054 (0.964, 1.154), 0.248566 |
| Current smoker | 1.192 (1.080, 1.316), 0.000514 | 1.163 (1.050, 1.289), 0.003893 | 1.158 (1.045, 1.283), 0.005175 |

Notes: Model 1 adjusted age, gender, race and BMI; Model 2 adjusted Model 1 plus marital status, education, family PIR and physical activity; Model 3 adjusted Model 2 plus serum uric acid, hypertension, coronary heart disease, diabetes and gout; Model 4 represented the effect values integrated after MI based on Model 3. Abbreviations: OR, Odds ratio; CI, Confidence interval.
