# Supplementary material for: Associations between smoke exposure and kidney stones: results from the NHANES (2007–2018) and Mendelian randomization analysis
Source: Front Med (Lausanne). 2023 Aug 10;10:1218051. doi: 10.3389/fmed.2023.1218051 (PMC10450509; doi:10.3389/fmed.2023.1218051)
Supplement: Supplementary Table S4 — The respective and pooled effect values after multiple imputations are based on serum cotinine concentrations. [file Table_4.DOCX]

**Supplementary** **Table S4.** The respective and pooled effect values after multiple imputation based on serum cotinine concentrations.

| **Serum cotinine concentrations** | **Model 1**  **OR (95% CI), *P*** | **Model 2**  **OR (95% CI), *P*** | **Model 3**  **OR (95% CI), *P*** |
| --- | --- | --- | --- |
| **Dataset 1** | | | |
| Log2-transformed serum cotinine (ng/ml) | 1.018 (1.011, 1.026),  < 0.00001 | 1.018 (1.010, 1.025),  < 0.00001 | 1.017 (1.010, 1.025),  < 0.00001 |
| Cotinine level category | | | |
| < 0.05 ng/ml | 1 | 1 | 1 |
| 0.05-2.99 ng/ml | 1.161 (1.050, 1.285), 0.00357 | 1.157 (1.044, 1.282), 0.00545 | 1.154 (1.041, 1.279), 0.00648 |
| ≥ 3.00 ng/ml | 1.253 (1.138, 1.379),  < 0.00001 | 1.241 (1.122, 1.372), 0.00003 | 1.234 (1.116, 1.365), 0.00004 |
| *P* for trend | < 0.001 | < 0.001 | < 0.001 |
| **Dataset 2** | | | |
| Log2-transformed serum cotinine (ng/ml) | 1.018 (1.011, 1.025),  < 0.00001 | 1.017 (1.010, 1.025),  < 0.00001 | 1.017 (1.009, 1.025), 0.00001 |
| Cotinine level category | | | |
| < 0.05 ng/ml | 1 | 1 | 1 |
| 0.05-2.99 ng/ml | 1.162 (1.050, 1.285), 0.00353 | 1.156 (1.043, 1.281), 0.00572 | 1.153 (1.040, 1.278), 0.00670 |
| ≥ 3.00 ng/ml | 1.251 (1.137, 1.377),  < 0.00001 | 1.238 (1.119, 1.369), 0.00003 | 1.231 (1.113, 1.362), 0.00005 |
| *P* for trend | < 0.001 | < 0.001 | < 0.001 |
| **Dataset 3** | | | |
| Log2-transformed serum cotinine (ng/ml) | 1.018 (1.011, 1.025),  < 0.00001 | 1.017 (1.010, 1.025),  < 0.00001 | 1.017 (1.009, 1.024), 0.00001 |
| Cotinine level category | | | |
| < 0.05 ng/ml | 1 | 1 | 1 |
| 0.05-2.99 ng/ml | 1.162 (1.050, 1.285), 0.00353 | 1.154 (1.042, 1.279), 0.00610 | 1.151 (1.039, 1.276), 0.00730 |
| ≥ 3.00 ng/ml | 1.252 (1.137, 1.378),  < 0.00001 | 1.235 (1.117, 1.366), 0.00004 | 1.228 (1.110, 1.358), 0.00007 |
| *P* for trend | < 0.001 | < 0.001 | < 0.001 |
| **Dataset 4** | | | |
| Log2-transformed serum cotinine (ng/ml) | 1.018 (1.011, 1.025),  < 0.00001 | 1.017 (1.010, 1.025),  < 0.00001 | 1.017 (1.010, 1.025),  < 0.00001 |
| Cotinine level category | | | |
| < 0.05 ng/ml | 1 | 1 | 1 |
| 0.05-2.99 ng/ml | 1.161 (1.050, 1.284), 0.00362 | 1.156 (1.044, 1.281), 0.00550 | 1.154 (1.041, 1.279), 0.00653 |
| ≥ 3.00 ng/ml | 1.251 (1.137, 1.377),  < 0.00001 | 1.239 (1.120, 1.370), 0.00003 | 1.232 (1.114, 1.363), 0.00005 |
| *P* for trend | < 0.001 | < 0.001 | < 0.001 |
| **Dataset 5** | | | |
| Log2-transformed serum cotinine (ng/ml) | 1.018 (1.011, 1.025),  < 0.00001 | 1.017 (1.014, 1.021),  < 0.00001 | 1.017 (1.009, 1.024), 0.00001 |
| Cotinine level category | | | |
| < 0.05 ng/ml | 1 | 1 | 1 |
| 0.05-2.99 ng/ml | 1.161 (1.114, 1.209),  < 0.00001 | 1.157 (1.109, 1.207),  < 0.00001 | 1.153 (1.040, 1.278), 0.00667 |
| ≥ 3.00 ng/ml | 1.252 (1.204, 1.302),  < 0.00001 | 1.240 (1.189, 1.292),  < 0.00001 | 1.229 (1.111, 1.359), 0.00006 |
| *P* for trend | < 0.001 | < 0.001 | < 0.001 |
| **Integration of effect values for five datasets** | | | |
| Log2-transformed serum cotinine (ng/ml) | 1.018 (1.011, 1.025), 0.000001 | 1.017 (1.010, 1.024), 0.000001 | 1.017 (1.009, 1.025), 0.000010 |
| Cotinine level category | | | |
| < 0.05 ng/ml | 1 | 1 | 1 |
| 0.05-2.99 ng/ml | 1.161 (1.059, 1.273), 0.001448 | 1.156 (1.053, 1.270), 0.002422 | 1.153 (1.040, 1.278), 0.006726 |
| ≥ 3.00 ng/ml | 1.252 (1.147, 1.366),  < 0.000001 | 1.239 (1.130, 1.358), 0.000005 | 1.231 (1.113, 1.361), 0.000055 |

Notes: Model 1 adjusted age, gender, race and BMI; Model 2 adjusted Model 1 plus marital status, education, family PIR and physical activity; Model 3 adjusted Model 2 plus serum uric acid, hypertension, coronary heart disease, diabetes and gout; Model 4 represented the effect values integrated after MI based on Model 3. Abbreviations: OR, Odds ratio; CI, Confidence interval.
