# Supplementary material for: Associations between smoke exposure and kidney stones: results from the NHANES (2007–2018) and Mendelian randomization analysis
Source: Front Med (Lausanne). 2023 Aug 10;10:1218051. doi: 10.3389/fmed.2023.1218051 (PMC10450509; doi:10.3389/fmed.2023.1218051)
Supplement: Supplementary Table S5 — Detailed information about screened-out SNPs. [file Table_5.DOCX]

**Supplementary** **Table S5.** Detailed information about screened out SNPs.

| **Exposure** | **Outcome** | **SNP** | **EA** | **NEA** | **EAF_exposure_** | **Beta_exposure_** | **SE_exposure_** | ***P*_exposure_** | **Beta_outcome_** | **SE_outcome_** | ***P*_outcome_** | **EAF_outcome_** | **F statistics** |
| --- | --- | --- | --- | --- | --- | --- | --- | --- | --- | --- | --- | --- | --- |
| Cotinine | KSD | rs114671050 | A | G | 0.011196 | 0.855589 | 0.166981 | 3.05e-07 | 0.19462 | 0.08931 | 0.0293 | 0.0118 | 26.25405 |
| Cotinine | KSD | rs117628878 | T | C | 0.013151 | -0.622631 | 0.129093 | 1.44e-06 | -0.03429 | 0.09427 | 0.7160 | 0.0107 | 23.26248 |
| Cotinine | KSD | rs13189249 | C | A | 0.290433 | -0.103840 | 0.021614 | 1.58e-06 | -0.00263 | 0.02175 | 0.9040 | 0.2690 | 23.08124 |
| Cotinine | KSD | rs142575813 | T | C | 0.018007 | -0.487692 | 0.102516 | 2.00e-06 | -0.03369 | 0.07631 | 0.6590 | 0.0175 | 22.63122 |
| Cotinine | KSD | rs2036527 | A | G | 0.348571 | 0.222835 | 0.020678 | 4.79e-27 | 0.02521 | 0.02048 | 0.2180 | 0.3350 | 116.13142 |
| Cotinine | KSD | rs2087352 | C | T | 0.191644 | 0.127258 | 0.025625 | 6.96e-07 | 0.01311 | 0.02367 | 0.5800 | 0.2180 | 24.66280 |
| Cotinine | KSD | rs2316205 | C | T | 0.508341 | -0.136647 | 0.020201 | 1.39e-11 | 0.02356 | 0.01948 | 0.2270 | 0.5460 | 45.75668 |
| Cotinine | KSD | rs2371107 | G | A | 0.722861 | -0.099986 | 0.021684 | 4.07e-06 | 0.00774 | 0.02215 | 0.7270 | 0.7440 | 21.26178 |
| Cotinine | KSD | rs294775 | T | C | 0.921953 | -0.354103 | 0.037300 | 2.38e-21 | -0.02435 | 0.03931 | 0.5360 | 0.9370 | 90.12423 |
| Cotinine | KSD | rs824892 | G | T | 0.598075 | 0.092692 | 0.020221 | 4.63e-06 | 0.03076 | 0.01977 | 0.1200 | 0.6010 | 21.01257 |

Abbreviations: SNPs, Single nucleotide polymorphisms; EA, Effect alleles; NEA, Non-effect alleles; EAF, Effect allele frequency; SE, Standard error.
