# Supplementary material for: Associations between smoke exposure and kidney stones: results from the NHANES (2007–2018) and Mendelian randomization analysis
Source: Front Med (Lausanne). 2023 Aug 10;10:1218051. doi: 10.3389/fmed.2023.1218051 (PMC10450509; doi:10.3389/fmed.2023.1218051)
Supplement: Supplementary Table S6 — Sensitivity analysis of the Mendelian randomization study. [file Table_6.DOCX]

**Supplementary** **Table S6.** Sensitivity analysis of Mendelian randomization study.

| **Exposure** | **Outcome** | **nSNPs** | **Heterogeneity** | | | **Pleiotropy test** | | **MR-Presso** |
| --- | --- | --- | --- | --- | --- | --- | --- | --- |
| Serum cotinine | KSD | 10 | Method | Q (df) | *P*-value | Egger-intercept | *P*-value | *P*-value |
|  |  |  | MR-Egger | 6.45 (8) | 0.596 | -0.0131 | 0.399 | 0.627 |
|  |  |  | IVW | 7.25 (9) | 0.611 |  |  |  |

Abbreviations: SNPs, Single nucleotide polymorphisms; MR-PRESSO, MR-Pleiotropy Residual Sum and Outlier; IVW, Inverse-variance weighting.
